# Risk factor screening to identify women requiring oral glucose tolerance testing to diagnose gestational diabetes: A systematic review and meta-analysis and analysis of two pregnancy cohorts

**DOI:** 10.1371/journal.pone.0175288

**Published:** 2017-04-06

**Authors:** Diane Farrar, Mark Simmonds, Maria Bryant, Debbie A. Lawlor, Fidelma Dunne, Derek Tuffnell, Trevor A. Sheldon

**Affiliations:** 1 Bradford Institute for Health Research, Bradford Institute for Health Research, Bradford Royal Infirmary, Bradford, United Kingdom; 2 Department of Health Sciences, University of York, York, United Kingdom; 3 Centre for Reviews and Dissemination, University of York, York, United Kingdom; 4 Leeds Institute of Clinical Trials Research, University of Leeds, Leeds, United Kingdom; 5 MRC Integrative Epidemiology Unit at the University of Bristol, Oakfield House, Oakfield Grove, Bristol, United Kingdom; 6 School of Social and Community Medicine, University of Bristol, Bristol, United Kingdom; 7 Galway Diabetes Research Centre (GDRC) and School of Medicine, National University of Ireland, Galway, Republic of Ireland; 8 Bradford Women’s and Newborn Unit, Bradford, United Kingdom; Florida International University Herbert Wertheim College of Medicine, UNITED STATES

## Abstract

**Background:**

Easily identifiable risk factors including: obesity and ethnicity at high risk of diabetes are commonly used to indicate which women should be offered the oral glucose tolerance test (OGTT) to diagnose gestational diabetes (GDM). Evidence regarding these risk factors is limited however. We conducted a systematic review (SR) and meta-analysis and individual participant data (IPD) analysis to evaluate the performance of risk factors in identifying women with GDM.

**Methods:**

We searched MEDLINE, Medline in Process, Embase, Maternity and Infant Care and the Cochrane Central Register of Controlled Trials (CENTRAL) up to August 2016 and conducted additional reference checking. We included observational, cohort, case-control and cross-sectional studies reporting the performance characteristics of risk factors used to identify women at high risk of GDM. We had access to IPD from the Born in Bradford and Atlantic Diabetes in Pregnancy cohorts, all pregnant women in the two cohorts with data on risk factors and OGTT results were included.

**Results:**

Twenty nine published studies with 211,698 women for the SR and a further 14,103 women from two birth cohorts (Born in Bradford and the Atlantic Diabetes in Pregnancy study) for the IPD analysis were included. Six studies assessed the screening performance of guidelines; six examined combinations of risk factors; eight evaluated the number of risk factors and nine examined prediction models or scores. Meta-analysis using data from published studies suggests that irrespective of the method used, risk factors do not identify women with GDM well.

Using IPD and combining risk factors to produce the highest sensitivities, results in low specificities (and so higher false positives). Strategies that use the risk factors of age (>25 or >30) and BMI (>25 or 30) perform as well as other strategies with additional risk factors included.

**Conclusions:**

Risk factor screening methods are poor predictors of which pregnant women will be diagnosed with GDM. A simple approach of offering an OGTT to women 25 years or older and/or with a BMI of 25kg/m^2^ or more is as good as more complex risk prediction models. Research to identify more accurate (bio)markers is needed.

Systematic Review Registration: PROSPERO CRD42013004608

## Introduction

Gestational diabetes mellitus (GDM) is hyperglycaemia of variable severity first identified in pregnancy. GDM is associated with an increased risk of a range of adverse perinatal outcomes,[1,2] including being born large (macrosomia) and there is growing evidence that the longer-term health of the mother and infant may be adversely affected.[3–5]

Treatment of GDM improves perinatal outcomes,[6–8] suggesting a role for identifying women with GDM. There is uncertainty about the effectiveness of different strategies for identifying these women, largely because of the lack of good quality evidence.[8,9] This has led to variation in clinical guidelines and practice for identifying GDM, both between and within countries. Strategies include selectively offering a 75g or 100g oral glucose tolerance test (OGTT) to high risk women only, identified using specific risk factors (usually easily identifiable maternal characteristics) or the administration of a 50g glucose challenge test. Alternatively all women can be offered an OGTT (universal offer of OGTT).[10,11] Restricting diagnostic testing to high risk women may be less costly than offering testing to all; the OGTT is relatively expensive and requires pregnant women to fast overnight and attend clinic for at least two hours. However, offering all women an OGTT may result in more women with GDM being identified and a reduction in adverse outcomes, as more affected women will receive treatment to reduce hyperglycaemia. GDM is also a risk factor for later development of type 2 diabetes,[3] if more women with GDM are identified, more could receive interventions aimed at reducing these risks and alongside lifelong screening to identify type 2 diabetes earlier, associated morbidities and costs may be reduced, however there is no robust evidence of longer-term benefit from the identification of GDM using a universal testing strategy.[8]

Risk factor screening involves the assessment of maternal characteristics, such as family history of diabetes; being of an ethnicity with a high prevalence of diabetes (i.e. non-white ethnicity: including Asian, black Caribbean or Middle Eastern); history of having GDM or a macrosomic infant; maternal obesity[12] and occasionally biochemical markers.[13,14]

Several healthcare agencies including the UK National Institute for Health and Care Excellence (NICE)[12], the American diabetes Association[15] and the Australasian Diabetes in Pregnancy Society (ADIPS)[16] recommend offering an OGTT to women with one or more risk factors (Table 1) in early pregnancy, some agencies then recommend repeat testing in this high risk group of women (those with risk factors), if GDM has not already been identified,[12] whilst others recommend all women (not previously identified as having GDM in early pregnancy) are offered an OGTT irrespective of risk factors.[16,17] For the majority of women the OGTT is conducted in mid-pregnancy (usually at 24–28 weeks gestation) so that the maximum number of women destined to develop hyperglycaemia will have a chance to be detected, while allowing enough time to provide treatment.

**Table 1:** Summary of selected screening strategies recommending the use of risk factors for the identification of gestational diabetes.

Agency	Nature of screening strategy
National Institute for Health and Care Excellence (UK NICE)[12] 2015	Offer women who have had GDM previously self-monitoring, blood glucose estimation or OGTT in early pregnancy.Offer OGTT at 24–28 weeks gestation only to women with at least one of:BMI >30kg/m^2^
	• Previous macrosomic baby (above 4.5kg)
	• Previous GDM
	• Family history of diabetes
	• Ethnic origin with a high prevalence of diabetes
American Diabetes Association(ADA)[17] 2017	Offer OGTT at first pregnancy visit to women who are overweight/obese (BMI≥25 kg/m2) or are Asian American and have at least one additional risk factor:
	• A1C ≥5.7% (39 mmol/mol), IGT, or IFG on previous testing
	• first-degree relative with diabetes
	• High-risk race/ethnicity (e.g., African American, Latino, Native American, Asian American, Pacific Islander)
	• Women who were diagnosed with GDM
	• History of CVD
	• Hypertension (≥140/90 mmHg or on therapy for hypertension)
	• HDL cholesterol level, 35 mg/dL (0.90 mmol/L) and/or a triglyceride level .250 mg/dL(2.82 mmol/L)
	• Women with polycystic ovary syndrome
	• Physical inactivity
	• Other clinical conditions associated with insulin resistance (e.g., severe obesity, acanthosis nigricans
	Test all women at 24 to 28 weeks gestation not previously known to have diabetes
Australasian Diabetes in Pregnancy Society (ADIPS)[16] 2014	Offer OGTT early in pregnancy to women who have a BMI ≥25kg/m^2^ or are from an ethnicity at high risk of diabetes (e.g. Asian, Aboriginal, Pacific Islander) and who have an abnormal fasting or random blood sugar Offer OGTT early in pregnancy to women with one of the risk factors below or who have both a BMI ≥25kg/m^2^ and are from an ethnicity at high risk of diabetes (e.g. Asian, Aboriginal, Pacific Islander)
	• Previous GDM
	• Previously elevated blood glucose level
	• Age ≥40 years
	• High-risk race/ethnicity
	• Family history of diabetes
	• Pre-pregnancy BMI > 35 kg/m2
	• Previous macrosomia
	• Polycystic ovarian syndrome
	• Medications: corticosteroids, antipsychotics
	Offer OGTT to all women at 24 to 28 weeks gestation not already identified as having GDM

IGT = impaired glucose tolerance test; IFG = impaired fasting glucose; BMI = body mass index; A1C = glycated haemoglobin; CVD = cardiovascular disease

Risk factor assessment is recommended in many populations in early pregnancy. The presence of a risk factor therefore influences early assessment of hyperglycaemia and whether mid-trimester testing in selectively tested populations is conducted. The aim of this study was to evaluate the performance of risk factors in identifying women requiring diagnostic testing for GDM, utilising published studies and available individual participant data.

## Methods

We conducted a systematic review and meta-analyses of published studies evaluating risk factors for the identification of women at high risk of GDM. The review was conducted in accordance with the Centre for Reviews and Dissemination’s guidance[18]. We also analysed individual participant data (IPD) from two large birth cohorts: Born in Bradford (BiB)[19] and Atlantic Diabetes in Pregnancy (Atlantic DIP)[20]. The methods and results are reported following the PRISMA guidelines (S1 File).[21]

### Search strategy

Title, abstract screening and then full text screening was performed in duplicate by two reviewers (DF, MS, SG or MB) with disagreements resolved by consensus or by a third reviewer.

### Search: identification of studies from the systematic review

Searches were undertaken up to August 2016 in MEDLINE, MEDLINE in-process, Embase, Maternity and Infant Care and CENTRAL with no date or country restrictions (S2 File). In addition to database searches, citation checking of included publications was undertaken.

### Study selection: Inclusion and exclusion criteria

All eligible published and on-going observational, cohort, case-control or cross-sectional studies were included. Due to time and cost constraints only studies published in English were included. Studies had to report data from women in whom risk factors for GDM were recorded and who were tested for GDM using an OGTT. We included studies that evaluated readily available/routinely collected maternal characteristics: age, ethnicity, parity, previous GDM, macrosomia, family history of diabetes, BMI and blood pressure. We did not include studies that focused solely on biochemical tests such as the 50g oral glucose challenge test, as these tests are less commonly used in universal pre-diagnostic test screening programmes and are more costly than risk factor screening.[12] We examined the value of using combinations of risk factors for selecting pregnant women for OGTT. Studies had to report the accuracy of combinations of risk factors; such as, numbers of risk factors present, risk models or scores based or measuring multiple risk factors, or the use of guideline recommendations. Studies reporting the screening accuracy of a single risk factor, without examining combinations of risk factors, were excluded.

No formal quality assessment process was undertaken because of the lack of any validated quality assessment tool for studies evaluating the performance of risk factors as a screening test; however studies had to report adequate information and that information had to be in a format that allowed comparison with others (described below in statistical analysis).

### Data extraction

Data were extracted by three reviewers (MS, SG, DF) and any disagreements resolved through discussion. Publication year, location, GDM diagnostic criteria, risk factors, cut-off levels of risk factors if appropriate and number of women included with risk factor combinations were recorded. The total number of women with and without GDM according to diagnostic test results and assessment of risk factor performance (sensitivity and specificity and positive predictive value, if reported) were recorded.

### Statistical analysis

For each group of risk factor combinations, sensitivity (proportion of GDM cases correctly identified by the risk factor); specificity (proportion of women without GDM correctly identified) and positive rate (proportion of women who would be offered an OGTT if the risk factor combinations were present) were calculated. Statistics were plotted for each study in Receiver Operating Characteristic (ROC) space, by plotting screening performance—sensitivity against positive rate.[22] A ‘good’ test will have high sensitivity with small numbers needing to be tested (with results near the top left of the space). Meta-analysis methods for pooling of screening studies, such as the Hierarchical summary receiver-operator curves (HSROC) model [23] were considered, but not performed because of the different screening approaches and included risk factors used by studies.

### Individual participant data (IPD) cohort analysis

Data from two birth cohorts were eligible and available. Born in Bradford (BiB) [19] is a prospective birth cohort (research ethics committee approval reference 07/H1302/112); the methods have been previously described.[19] The Atlantic Diabetes in Pregnancy study (Atlantic DIP) is a multi-centre cohort study comprising of a partnership of five hospitals at the Irish Atlantic seaboard (research ethics committee approval was obtained from participating centres); study methods have been previously described.[20] Both cohorts offered all women a 75g OGTT irrespective of the presence of risk factors. The World Health Organization (WHO) 1999 (modified) criteria were used to diagnose GDM (fasting glucose ≥6.1mmol/l, two-hour post-load glucose ≥7.8mmol/l) in both cohorts.[24,25]

### Statistical analysis

Risk factors recorded by the IPD cohorts were similar to those recorded by published studies included in the systematic review. We considered seven commonly used risk factors: age; BMI; parity (multiparous, primiparous); ethnicity (White, South Asian or Other), family history of diabetes; previous GDM or having had a previous macrosomic infant. We grouped women into white (British/Irish), south Asian or other, as these groupings most appropriately represent the ethnicities of the women in the included cohorts, it should be noted that these groupings may not be appropriate for other populations. Data on previous GDM or having had a previous macrosomic infant were not available in the Atlantic DIP cohort.

We classified BMI using the thresholds of 25kg/m^2^ (kilogramme/meter^2^) or over, or 30kg/m^2^ or over; because these are the recommended thresholds for overweight and obesity.[26,27] We used the age categories of 25 years or older, or 30 years or older, because they have been used previously [28–31] and are clinically relevant. This generated 287 combinations of risk factors. The sensitivity, specificity and positive rate were calculated for each combination of risk factors and those that were “dominated” by another in that class (i.e. a combination is dominated if there is one other related ‘test’ with both higher sensitivity and specificity which would be a better predictor) were removed. Sensitivity and positive rates for the remaining non-dominated tests were plotted in ROC space.

In addition we also examined screening performance based on a predicted risk of GDM, similar to screening strategies used to identify those at risk of cardiovascular disease.[32] A logistic regression model was fitted to data from each of the cohorts and to a pooled cohort dataset for comparison, regressing GDM incidence against the seven included risk factors. The resulting log odds ratios were used to calculate a predicted risk of GDM for each woman in the dataset. The sensitivity and positive rate for predicting GDM at each percentage point of risk from 1% to 80% was calculated and plotted in ROC space.

## Results

### Systematic review and meta-analysis

**Fig 1:**
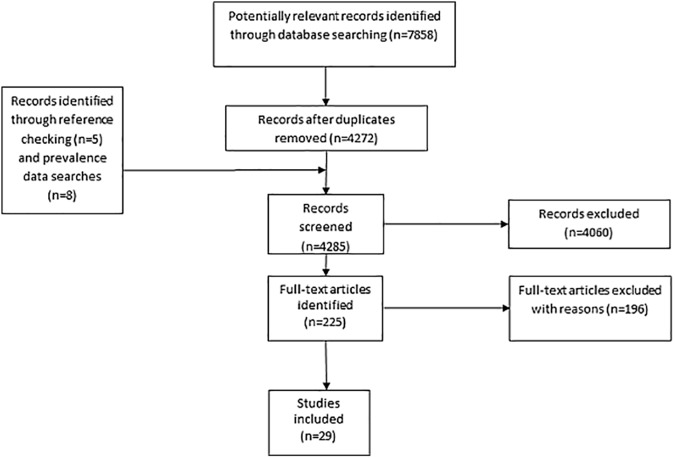
Flow chart of the systematic review search process.

Searches identified 4272 unique citations (7858 before de-duplication). Thirteen additional publications were identified through reference checking. After title and abstract screening, 225 publications were retrieved for full-text screening. One hundred and ninety six full text papers were excluded because they did not meet eligibility criteria, leaving 29 studies (Fig 1), with 211,698 women. Six studies [33–38] assessed the screening performance of guideline recommendations (UK National Institute for Health and Care Excellence (NICE),[37] American Diabetes Association (ADA),[35–38] American College of Obstetricians and Gynecologists (ACOG),[36] Australasian Diabetes In Pregnancy Society (ADIPS),[37] Irish,[33] French[34]). Eight studies evaluated the screening performance of the number of risk factors (for example if two, three or four etc. risk factors were present),[39–46] six examined combinations of risk factors[28,47–51] and nine studies examined the ability of a risk prediction model or a risk score to predict GDM. [52–60]

All studies were observational, consisting of a mix of prospective and retrospective cohort studies, with GDM diagnosed using an OGTT, using specified diagnostic criteria. Diagnostic criteria and glucose thresholds varied between studies, which influenced estimates of GDM prevalence. Studies were diverse in their included populations (Table 2).

**Table 2:** Characteristics of studies included in the systematic review.

First author	Year	Country	GDM diagnosis criterion	Total women	No. with GDM	% with GDM	Risk factor screening strategy
Avalos[33]	2013	Ireland	IADPSG	5500	521^a^	9	Irish, NICE, ADA guideline recommendations
					491^b^	9	
					585^c^	11	
Caliskan[39]	2004	Turkey	NDDG	422	14	3	Number of risk factors
Cosson[34]	2013	France	WHO	18755	2710	14	French guideline recommendations
Cypryk[40]	2008	Poland	WHO	2180	510	23	Number of risk factors
Danilenko-Dixon[35]	1999	USA	NDDG	18504	564	3	ADA guideline recommendations
Erum[51]	2015	Turkey	ADA	815	39	5	‘At least one risk factor’
Gabbay-Benviz[58]	2015	USA	C&C	924	63	7	Risk score
Jensen[61]	2003	Denmark	DPSG	2992^d^	83	3	Number of risk factors
Jiminez-moleon[36]	2002	Spain	NDDG	1436	58^c^	4	ADA and ACOG guideline recommendations
				2174	63^e^	3	
Kirke[59]	2014	Australia	WHO	1636	73	4	Risk score
Marquette[42]	1985	USA	C&C	434	12	3	Number of risk factors
Moses[47]	1998	Australia	ADIPS	2907	183	6	Age, BMI ethnicity
Nanda[52]	2011	UK	WHO	11464	297	3	Risk model
Naylor[53]	1997	US	NDDG or C&C	1571	69	4	Risk score
Nielsen[43]	2016	India	WHO	3946	659	17	Number of risk factors (1, 2 or 3)
Ostlund[28]	2003	Sweden	WHO	3616	61	5	"Traditional risk factors"
Phaloprakam[54]	2009	Thailand	C&C	469	127	27	Risk score
Pintaudi[48]	2014	Italy	IADPSG	1015	113	11	"Standard risk factors"
Sacks[44]	1987	USA	ADA	4116	138	3	Number of risk factors
Savona-Ventura[49]	2013	Mediterranean	ADA	1368	119	9	Based on age, obesity or diastolic BP
Shamsuddin[45]	2001	Malaysia	OGTT levels reported	768	191	25	Number of risk factors
Shirazian[55]	2009	Iran	ADA	924	68	7	Risk score
Sunsaneevithayakul[46]	2003	Thailand	Not reported	9325	235	2	Number of risk factors
Syngelaki[60]	2015	UK	WHO	75161	1827	20	Risk model
Teh[37]	2011	Australia	ADIPS	2426	250	10	NICE, ADA and ADIPS guideline recommendations
van Leeuwen[57] (A)	2010	Netherlands	OGTT/GCT levels reported	995	24	2	Risk model
van Leeuwen[56] (B)	2009	Netherlands	WHO	1266	47	4	Risk score
Williams[50]	1999	USA	NDDG	25118	210^f^	1	Based on age, BMI ethnicity, family history
Yang[38]	2002	China	WHO	9471	171	2	ADA guideline

^a^Irish guideline

^b^NICE guideline

^c^ADA recommendations

^d^Jensen (2003), 5235 women were included in the study, 2992 had an OGTT performed

^e^ACOG recommendations

^f^Williams (1999), number of women with GDM varied by the recorded risk factor (i.e. not all women had all risk factors recorded)

ACOG = American College of Obstetricians and Gynecologists

ADA = American Diabetes Association

ADIPS = Australasian Diabetes In Pregnancy Society

C&C = Carpenter and Coustan

NDDA = National Diabetes Data Group

NICE = National Institute for Health and Care Excellence

IADPSG = International Association of Diabetes in Pregnancy Study Groups

WHO = World Health Organization

### Performance of risk factors in predicting GDM

**Fig 2:**
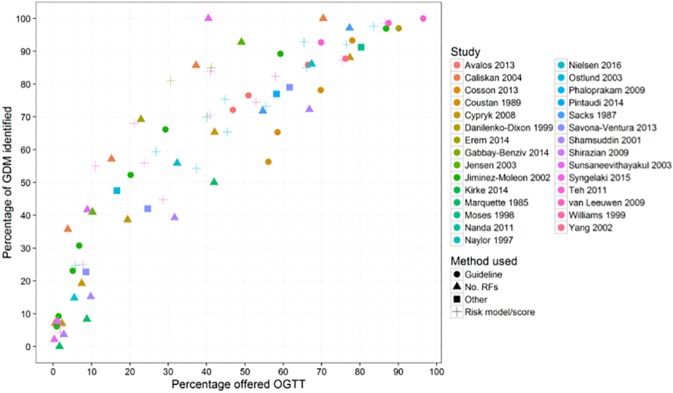
Screening performance (sensitivity and percentage offered an oral glucose tolerance test (OGTT)) by study and by risk factor method (guideline recommendations, number (No) of risk factors, ‘other method and risk model/score). The colour of the points indicates the study. The shape of the points (circles, triangle, square, cross) indicates method used No. RF = number of risk factors (i.e. presence of one risk factor, two risk factors and so on). Studies may report more than one performance estimate, this is reflected in the number of coloured shapes for each study.

**Fig 3:**
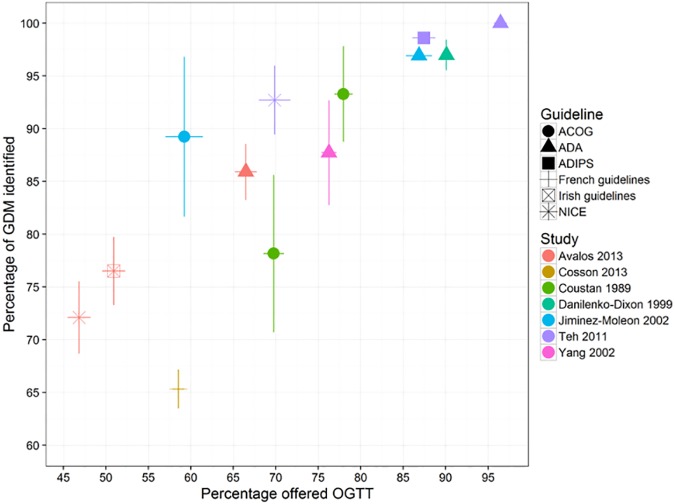
Screening performance of guidelines using a risk factor screening strategy. Vertical and horizontal lines show the 95% confidence intervals for sensitivity and positive rate respectively. The colour of the points indicates the study. The shape of the points (circles, triangle, square, cross) indicates method used. RF = Risk factor, No = number. ACOG = American College of Obstetricians and Gynecologists. ADA = American Diabetes Association. ADIPS = Australasian Diabetes In Pregnancy Society. NICE = National Institute for Health and Care Excellence. Studies may report more than one performance estimate, this is reflected in the number of coloured shapes for each study.

**Fig 4:**
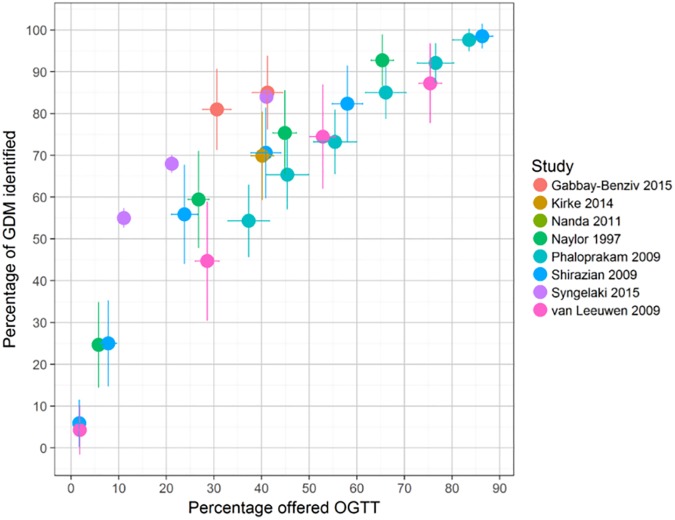
Screening performance of risk prediction or scoring models. The colour of the points indicates the study. Vertical and horizontal lines show the 95% confidence intervals for sensitivity and positive rate respectively. Studies may report more than one performance estimate, this is reflected in the number of coloured shapes for each study

Figs 2 to 4 show estimates of sensitivity and proportion of women that would be offered an OGTT for each of the included studies, plotted in the ROC space. Fig 2 includes data from all 29 studies and shows, as one would expect, that the proportion of correctly identified GDM cases (sensitivity) increases with the number of women offered an OGTT, irrespective of the risk factor strategy used, there seems to be no obvious ‘best’ approach.

Fig 3 shows the proportion of correctly identified GDM cases and proportion offered an OGTT for different screening recommendations (American College of Obstetricians and Gynecologists (ACOG), American Diabetes Association (ADA), Australasian Diabetes in Pregnancy Society (ADIPS) and the UK National Institute for Health and Care Excellence (NICE)). There is considerable variation in both sensitivity and number of women offered an OGTT. The screening performance of guideline recommendations appears moderate at best, because generally at least 70% of women would need to be offered an OGTT to identify 80% of all women with GDM, with the exception of the ACOG guideline when applied to an Irish[33] or Spanish[36] population and the ADA guideline when applied to an Irish population.[33]

Fig 4 shows the results from eight studies that examined the sensitivity and number of women offered an OGTT after the application of a risk prediction model or risk score. [52–57] Each study has several points on the ROC curve because results are reported for various levels of risk. Results are reasonably consistent across studies with all points generally lying on a similar ROC curve.

Figs 2 to 4 clearly show a trade-off; as sensitivity increases (and more women are identified), the number needed to receive a diagnostic test also increases. For example Fig 4 shows that to identify 80% of women with GDM (sensitivity of 80%) using a risk prediction model or risk score, between 30% and 58% of women would need to undergo an OGTT (depending which risk model is used); to achieve a sensitivity of over 90%, nearly all women would need to undergo an OGTT.

### Individual participant data analysis

#### Screening based on combinations of risk factors

**Fig 5:**
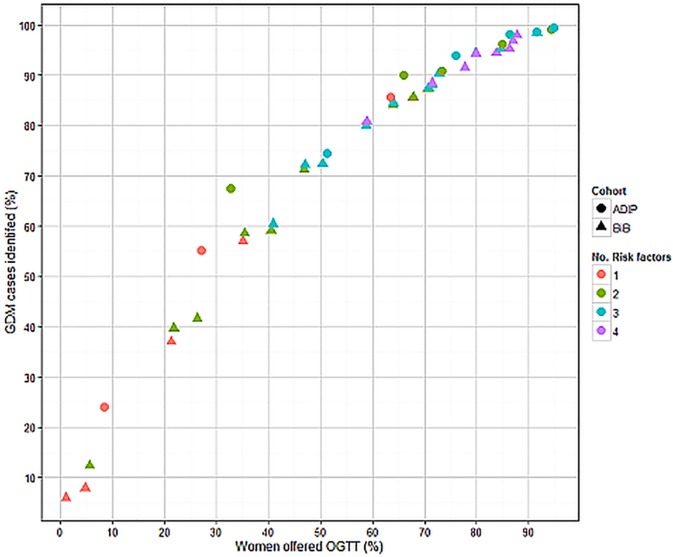
Screening performance of risk factor combinations for identifying GDM using IPD. The colour of the points indicates the number (No) of risk factors included. Circles indicate results for Atlantic DIP and triangles represent results for BiB.

Fig 5 shows the percentage of GDM cases identified (sensitivity) against percentage of women offered an OGTT (positive rate) for each group of risk factors not ‘dominated’ by others. Irrespective of the number of risk factors included (one risk factor through to the use of four); all groups generally lay on the same ROC curve.

Fig 5 shows that using multiple risk factors is not superior to using just one or two, because the increase in sensitivity is only achieved by increasing the number of women offered an OGTT. Both cohorts demonstrate generally similar estimates of sensitivity and positive rate for each number of risk factors.

Table 3 shows examples of the performance of combinations of risk factors (two, through to four, not dominated) with sensitivity between 90% and 95% (detecting almost all cases of GDM) and for the UK NICE guideline recommended group of risk factors.[12] A woman is test positive (and therefore would be offered an OGTT) if she has one or more of the named risk factors in each group. Combining risk factors to produce the highest sensitivities, results in low specificities (and so higher false positives). Strategies that use only age and BMI categories however, perform similarly to others with additional risk factors. In our analyses, the NICE guideline recommended risk factor strategy was dominated by other strategies (other strategies had superior performance). For example, using combined cohort data, screening based on being either 25 years or older or having a BMI of 30 or over, achieved a higher sensitivity than using the combined NICE guideline recommended risk factors (Table 1) (93.2% and 78.2% respectively), but with a correspondingly higher positive rate (78.0% and 67.2% respectively) and lower specificity (23.3% and 31.7% respectively).

**Table 3:** Performance of risk factors, grouped by age, BMI and UK NICE categories for the identification of GDM using IPD

Risk factors	Sensitivity	Specificity	Positive rate
***BiB cohort***			
Age≥25 BMI≥30	90.4	28.7	72.7
Age≥25 BMI≥30, prior GDM	90.4	28.6	72.8
Age≥25 BMI≥30, FH of diabetes	91.6	23.2	77.7
Age≥25 BMI≥30, FH of diabetes, prior GDM	91.6	23.1	77.7
Age≥30, BMI≥30, non-white ethnicity	94.3	21.3	79.8
Age≥30, BMI≥30, non-white ethnicity, prior GDM	94.3	21.3	79.9
Age≥25, BMI≥25, FH of diabetes	94.4	16.9	83.8
Age≥25, BMI≥25, FH of diabetes, prior GDM	90.4	28.7	72.7
***Atlantic DIP cohort***^a^			
BMI≥25, non-white ethnicity	90.1	36.8	66.0
Age≥30, BMI≥30	90.8	28.6	73.4
Age≥30, BMI≥30, non-white ethnicity	93.9	26.0	76.0
***Cohorts combined***			
Age≥30, BMI≥30, FH of diabetes	90.0	24.6	76.4
Age≥30, BMI≥25, FH of diabetes, prior GDM	90.3	24.6	76.5
BMI≥25, non-white ethnicity	92.0	24.0	77.3
BMI≥25, non-white ethnicity, prior GDM	92.1	24.0	77.3
Age≥25, BMI≥30	93.2	23.3	78.0
Age≥25, BMI≥30, prior GDM	93.2	23.3	78.1
Age≥30, BMI≥30, non-white ethnicity	94.1	22.7	78.7
Age≥30, BMI≥30, non-white ethnicity, prior GDM	94.1	22.7	78.7
Age≥25, BMI≥25	95.9	16.5	84.5
Age≥25, BMI≥25, prior GDM	95.9	16.5	84.5
NICE guideline recommended risk factors[12]	78.2	31.7	67.2

BMI = body mass index (kg/m^2^)

FH = family history

NICE = National Institute for Health and Care Excellence

^a^Previous macrosomia and GDM not available in Atlantic DIP

#### Screening using risk prediction models

The odds ratios for the association between each risk factor and GDM for each cohort are shown in Table 4. All risk factors examined, apart from multiparity, were positively associated with GDM.

**Table 4:** The associations between risk factors and GDM using IPD

	BiB		Atlantic DIP	
	Odds ratio	95% Confidence interval	Odds ratio	95% Confidence interval
**Risk factor** Age (per year)	1.09	1.08 − 1.1	1.10	1.07 − 1.12
BMI (per kg/m^2^)	1.06	1.05 − 1.08	1.13	1.11 − 1.15
Ethnicity (non-white)	2.32	1.90 − 2.83	5.16	3.85 − 6.91
Multiparity	0.89	0.73 − 1.08	0.74	0.58 − 0.96
Family history of diabetes	1.36	1.14 − 1.63	1.42	1.17 − 1.80
Previous macrosomia^a^	1.54	1.12–2.13	-	-
Previous GDM^a^	5.90	3.78–9.22	-	-

^a^not available in Atlantic DIP

When considering risk factors available in both cohorts, the odds ratios were generally consistent, with the exception of non-white/Irish ethnicity, the strength of the association being more than twice that in Atlantic DIP than BiB (half the participants in BiB are of south Asian (non-white) origin, half are white British, whereas few women in Atlantic DIP are non-white). ‘Having had GDM in a previous pregnancy’ was most strongly associated with GDM in BiB (this risk factor was not available in Atlantic DIP).

**Fig 6:**
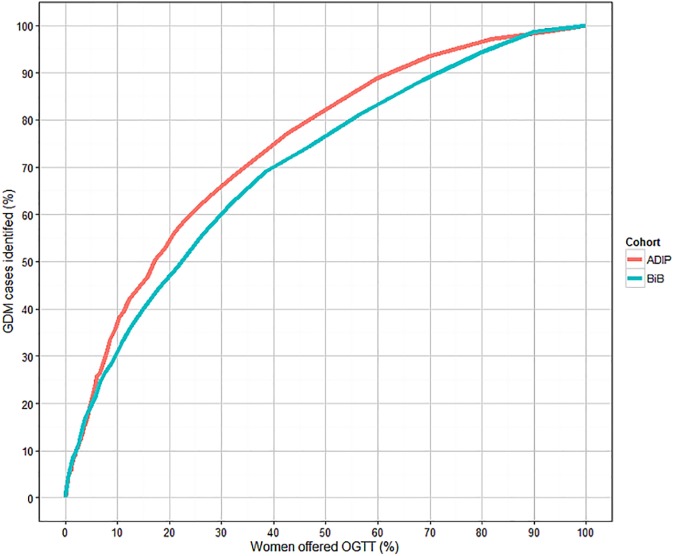
Sensitivity and positive rate when using a risk prediction model to predict GDM using IPD

The odds ratios shown in Table 4 were used to construct a predicted risk of GDM for each woman in each cohort. The ROC curves of sensitivity against positive rate are shown in Fig 6 and are similar for the two cohorts, though the performance seems marginally better for Atlantic DIP compared to BiB. The areas under the curves (AUCs) being 0.77 for Atlantic DIP and 0.72 for BiB, suggesting modest screening performance. Performance using a predictive risk model (Fig 6) seems similar to using a combination of several risk factors.

## Discussion

To our knowledge, this is the first systematic review and meta-analysis to assess the predictive accuracy of different combinations of risk factors to identify women at high risk of GDM. We found that universal risk factor pre-diagnostic test screening can take a variety of forms, but whatever the form, this strategy did not appear effective for accurately identifying women with GDM. Furthermore we found no evidence that complex risk screening strategies using several risk factors or risk prediction models offered significant benefit over the simpler strategy of identifying one or two risk factors. Regardless of the methods used, correctly identifying most women with GDM, requires offering an OGTT to the majority of women and therefore does not vary considerably from offering all women an OGTT. For some populations however, limiting the offer of an OGTT to high risk women may result in important cost savings.

Our IPD analyses suggest that the risk factor combination of maternal age and BMI (25 years or older and BMI ≥25 kg/m^2^) would identify the majority of women with GDM, but consistent with our systematic review findings, would mean inviting most women for an OGTT. Although this is as effective as more complex strategies (risk prediction models for example) it may not vary greatly from offering all women an OGTT.

### Strengths and limitations

This study examined published data identified by a systematic search, comprising 29 studies and including 211,689 women. We also conducted analyses using IPD from two large contemporary birth cohorts including 14,103 women. The findings from the published studies and IPD cohorts were consistent with each other. As well as triangulating findings from these two different designs we also compared findings from two different analytical approaches and also found consistency there, suggesting that our results are robust. Different populations based on geography and age were included suggesting that our results might be broadly generalisable to different antenatal populations in high income countries. Very few studies were from low income countries and it is therefore important to note that our findings may not generalise to those countries. Given the increase in non-communicable diseases in low and middle income countries and the scarcity of resources to be able to adequately deal with them, there is clearly a need to gain better understanding about how to screen for, diagnose and treat GDM in those countries.

Recommendations regarding the identification of GDM vary and some institutions that previously recommended risk factor assessment now recommend offering all women an OGTT, however there is a lack of supporting evidence that this strategy improves maternal and offspring health compared to selective testing high risk women [8] and given the likely increase in associated costs, clinicians and commissioners may not be willing or able to accept universal testing for GDM. The risk factors that we were able to assess in published studies were limited by what was available, but they included a range of the commonly used risk factors for GDM. Studies used varying threshold criteria and this influences the numbers of women identified by risk factors and makes comparison complex. Applying the same criteria in dissimilar populations however will also produce varying results (see the NICE guideline results in Fig 3 and Table 3). A more consistent global approach to identifying women with GDM would reduce variation in practise and would likely improve care. Although our search did not identify any; it is possible that there may be eligible studies published in languages other than English.

### Conclusions and implications for practice

Our results suggest that pre-diagnostic risk factor screening is a poor method for identifying women with GDM. Using this strategy will reduce the likely impact of antenatal GDM screening, testing and management programmes. Given these findings, there is an need for research to develop and evaluate (bio)markers that might more accurately identify women at high and low risk of GDM. Until then and if universal offer of an OGTT is not adopted, our results suggest that using age with a cut-off of 25 years (i.e. referring women at or older than 25 years for an OGTT) or who have a BMI of ≥25 or ≥30 kg/m^2^ would be currently the simplest and most accurate risk factor screening method. Ultimately though, the choice of whether and how to identify GDM should be informed by rigorous cost-effectiveness analysis.

## Supporting information

S1 File PRISMA 2009 checklist. (PDF)

S2 File Medline search strategy. (PDF)
