# Supplementary material for: Risk factor screening to identify women requiring oral glucose tolerance testing to diagnose gestational diabetes: A systematic review and meta-analysis and analysis of two pregnancy cohorts
Source: PLoS One. 2017 Apr 6;12(4):e0175288. doi: 10.1371/journal.pone.0175288 (PMC5383279; doi:10.1371/journal.pone.0175288)
Supplement: S2 File — (PDF) [file pone.0175288.s002.pdf]

S1: Medline search strategy

1. risk/ (98,061)
2. risk factors/ (563,466)
3. risk$.tw. (1,321,838)
4. related.tw. (1,576,712)
5. relationship.tw. (681,818)
6. rates.tw. (688,903)
7. difference$.tw. (1,634,999)
8. prevalence.tw. (382,911)
9. associated factors.tw. (8200)
10. predict$.tw. (943,185)
11. or/1-10 (5547,109)
12. exp overweight/ (142,733)
13. (obese or obesity).tw. (175,969)
14. (overweight or over weight).tw. (38,413)
15. body mass index/ (80,128)
16. BMI.tw. (75,276)
17. body mass index.tw. (101,816)
18. exp Ethnic Groups/ (112,019)
19. (ethnicity or ethnic or multiethnic$ or race).tw. (133,942)
20. (caucasian$ or asian$ or spanish or mexican$ or hispanic$ or afrocaribbean$ or african$ or caribbean$).tw.)
21. (237,285)
22. (middle eastern or bangladeshi$ or pakistani$).tw. (6067)
23. maternal age/ (16,131)
24. age.tw. (1,482,617)
25. (pregnan$ adj2 late$ adj2 life).tw. (41)
26. older.tw. (263,039)
27. over 35.tw. (2427)
28. over 25.tw. (3623)
29. over 30.tw. (10,584)
30. (previous adj3 (gdm or diabet$)).tw. (2130)
31. (prior adj3 (gdm or diabet$)).tw. (1392)
32. (history adj3 (gdm or diabet$)).tw. (7728)
33. (family adj3 (gdm or diabet$)).tw. (3296)
34. (relative adj3 (gdm or diabet$)).tw. (1141)
35. family history.tw. (42,205)
36. prior history.tw. (3983)
37. previous history.tw. (7267)
38. ((prior or previous or history) adj2 macrosomia).tw. (48)
39. ((prior or previous or history) adj2 macrosomic).tw. (25)
40. ((prior or previous or history) adj2 LGA).tw. (5)
41. ((prior or previous or history) adj2 large gestational age).tw. (0)
42. ((prior or previous or history) adj2 large for gestational age).tw. (1)
43. ((prior or previous or history) adj2 large bab$).tw. (3)
44. ((prior or previous or history) adj2 large infant$).tw. (2)
45. parity.tw. (21,039)
46. parity/ (20,499)
47. risk factor$.ti. (73,764)
48. or/12-46 (2,152,353)

49. 11 and 47 (1,297,220)
50. exp diabetes, gestational/ (6917)
51. (gestation$ adj4 diabet$).tw. (7955)
52. gdm.tw. (2973)
53. (glucose adj4 (pregnan$ or gestation$ or prenatal$ or antenatal$ or pre-natal$ or ante-natal$ or maternal$)).tw.)
54. (3380)
55. exp Hyperglycemia/ (26,216)
56. exp Pregnancy/ (711,357)
57. 53 and 54 (1565)
58. ((hyperglycemi$ or hyperglycaemi$) adj5 (pregnan$ or gestation$ or prenatal$ or antenatal$ or pre-natal$ or)
59. ante-natal$ or maternal$)).tw. (884)
60. or/49-52,55-56 (13,211)
61. 48 and 57 (5062)
62. Mass Screening/ (81,803)
63. screen$.ti. (116,932)
64. screen$.ab. /freq=2 (122,782)
65. Glucose Tolerance Test/ (29,299)
66. Blood glucose/an (47,809)
67. (glucose adj3 (test$ or measur$ or assess$ or evaluat$)).tw. (36,335)
68. ((glucose adj2 tolerance) or gtt or ogtt).tw. (33,566)
69. ((glucose adj2 challeng$) or gct or ogct).tw. (4698)
70. (fasting adj2 glucose).tw. (24,915)
71. or/59-67 (332,758)
72. Diagnosis/ (16,639)
73. Prenatal Diagnosis/ (31,200)
74. exp Diagnostic errors/ (94,398)
75. Diagnosis, Differential/ (379,054)
76. diagnos$.ti. (450,817)
77. diagnos$.ab. /freq=2 (536,082)
78. (di or du).fs. (2,229,471)
79. exp “Sensitivity and Specificity”/ (416,076)
80. (sensitivity or specificity).tw. (725,114)
81. ((pre-test or pretest) adj probabilit$).tw. (1402)
82. ((post-test or posttest) adj probabilit$).tw. (738)
83. (predictive adj3 value$).tw. (70,018)
84. (false positiv$ or false negativ$).tw. (56,289)
85. observer variation$.tw. (959)
86. roc curve$.tw. (14,358)
87. (likelihood adj3 ratio$).tw. (9220)
88. accurac$.tw. (232,990)
89. detection.tw. (591,376)
90. or/69-86 (3,919,528)
91. 68 or 87 (4,099,746)
92. 58 and 88 (2801)
93. animals/ not humans/ (3,855,883)
94. (editorial or case reports or news or letter or comment).pt. (2,995,254)
95. 89 not (90 or 91) (2665)
96. limit 92 to english language (2429)
